# Synthetic Peptides Suppress Nervous Necrosis Virus Absorption and Improve Survival Rates in European Sea Bass

**DOI:** 10.1007/s10126-025-10507-z

**Published:** 2025-08-23

**Authors:** Alberto Cuesta, Francisco J. Fernández-Hernández, Ana C. Hernández-Sendra, Constanza Cárdenas, Fanny Guzmán, Yulema Valero

**Affiliations:** 1 Immunobiology for Aquaculture Group, Department of Cell Biology and Histology, Faculty of Biology, University of Murcia, 30100 Murcia, Spain; 2 Núcleo Biotecnología Curauma (NBC), Pontificia Universidad Católica de Valparaíso, Valparaíso, Chile

**Keywords:** Betanodavirus, Synthetic peptides, Viral cycle, European sea bass, Adsorption, Replication

## Abstract

With few preventive strategies available against nodavirus (NNV) in aquaculture, therapeutic applications remain underexplored. This study aimed to peptide-based treatments disrupting critical stages of its viral life cycle. Thus, we designed and synthesized seven low-molecular-weight peptides (P1–P7) based on predicted binding regions of the capsid protein from the red-spotted grouper nervous necrosis virus (RGNNV) genotype to mimic viral capsid regions. Although in silico predictions suggested limited direct antiviral activity, in vitro assays using the E-11 cell line and in vivo trials in RGNNV-infected European sea bass (*Dicentrarchus labrax*) juveniles yielded promising results. The peptides, particularly when co-administered individually or as P3 + P4 and P5 + P6 combinations with the virus, disrupted RGNNV attachment in vitro. Moreover, they exhibited cross-reactivity against the striped jack nervous necrosis virus (SJNNV) genotype and both RGNNV/SJNNV and SJNNV/RGNNV reassortants. Treatment of RGNNV-infected sea bass significantly increased the relative percent survival, ranging from 81.3% for P4 to 62.5% for P3 and P3 + P4, while reducing viral load within 48 h post-treatment without altering systemic antiviral immune responses, tested through the transcriptional levels of *mx* gene in the head-kidney. Notably, peptide P4 partially inhibited viral replication in vitro at the same time-point when cells were pre-treated for 24 h, likely through modulation of host immune responses. These findings highlight the potential of targeted peptide-based therapies as a promising antiviral therapeutic strategy against NNV infections.

**Supplementary Information:**

The online version contains supplementary material available at 10.1007/s10126-025-10507-z.

## Introduction

Fish nervous necrosis virus (NNV; family *Nodaviridae*, genus *Betanodavirus*) is a segmented, non-enveloped RNA virus with an icosahedral structure, comprising two positive-sense single-stranded RNAs, RNA1 and RNA2, encapsulated by 180 copies of a single capsid protein (Chen et al. 2015; Liu et al. 2005). RNA1 encodes a 110 kDa RNA-dependent RNA polymerase (RdRp, so-called protein A), essential for genome replication. A subgenomic RNA3 also arises from RNA1, producing proteins B1, which exhibits anti-necrotic activity (Chen et al. 2009), and B2, which suppresses RNA silencing (Fenner et al. 2006; Iwamoto et al. 2005). RNA2 encodes a 42 kDa capsid protein (CP, so-called protein alpha (α)), required for capsid assembly and dependent on RNA1 for replication (Chen et al. 2015; Chérif et al. 2010). The CP comprises the N-ARM, S-domain, linker, and P-domain. N-ARM is critical for assembly, RNA encapsulation, intracellular targeting, and threefold symmetry axis formation (Guo et al. 2004, Chen et al. 2015). The S-domain, with a jelly-roll structure, ensures capsid stability, RNA encapsulation, receptor binding, trimerization, and pH-dependent infectivity (Chen et al. 2015; Štěrbová et al. 2024). The linker domain connects the S- and P-domains, providing flexibility for these interactions (Chen et al. 2015). The P-domain mediates host-cell binding, immune recognition, endocytosis, and pH-dependent infectivity (Chen et al. 2015; Štěrbová et al. 2024). Together, these regions ensure efficient assembly, stability, and host interaction (Chen et al. 2015; Štěrbová et al. 2024).

NNV causes viral encephalopathy and retinopathy (VER), affecting nearly 200 fish species, mainly at larval and juvenile stages. Its four parental strains—red grouper (RGNNV), striped jack (SJNNV), barfin (BFNNV), and tiger puffer (TPNNV)—differ in temperature and host preferences having global distribution (Ito et al. 2008; Nishizawa et al. 1997; Bandín and Souto 2020). Natural recombinants RGNNV/SJNNV and SJNNV/RGNNV, classified by RNA1/RNA2 origins, were identified in the Mediterranean Sea (Valero and Cuesta 2023), where European sea bass (*Dicentrarchus labrax*) and gilthead seabream (*Sparus aurata*) dominate aquaculture production (FAO 2024). Sea bass is highly susceptible to RGNNV, suffering severe outbreaks and high mortalities during early development (LeBreton et al. 1997). Conversely, seabream typically acts as an asymptomatic carrier (Castric et al. 2001). However, seabream early larvae, but not sea bass, have recently shown susceptibility to reassortant strains during natural outbreaks (Volpe et al. 2020) and laboratory trials (Volpe et al. 2020; Chen et al. 2015; García-Álvarez et al. 2022), challenging previously accepted infection tropisms (Valero and Cuesta 2023), further complicating aquaculture management in the region.

NNV remains a major concern in aquaculture, as experimental preventive measures have failed to prevent its spread (Padrós et al. 2022; Bandín and Souto 2020). Current strategies rely on good husbandry, focusing on sanitation and biosecurity (Costa and Thompson 2016; Doan et al. 2017), preventing the introduction of viral reservoirs through eggs and larvae, and selecting NNV-free broodstock (Doan et al. 2017). Research also focuses on in vivo preventive tools, including vaccines (Barsøe et al. 2021; Thwaite et al. 2020; Vázquez-Salgado et al. 2024; López-Vázquez et al. 2022; Valero et al. 2024, 2023, 2016), antimicrobial peptides (AMPs) (Valero et al. 2021a; Cervera et al. 2024a; Wang et al. 2010) or antivirals (Huang et al. 2016; Zhu et al. 2022). Despite these efforts, survival improvements remain limited, with only two commercial vaccines available. Therapeutic studies, including AMPs and antivirals, have achieved limited success (Cervera et al. 2024b, 2025; Zhu et al. 2022), and no commercial treatments exist to date.

Taken together, these findings highlight the urgent need for alternative therapies. This study designed short peptides targeting the RGNNV life cycle. Using its capsid as a template, short peptides corresponding to potential binding regions of the RGNNV capsid were synthetised and used to compete with the viral CP. All peptides showed strong in vitro antiviral activity against RGNNV by disrupting absorption, exhibiting cross-activity against SJNNV and reassortant strains. Most peptides significantly reduced mortality and disease symptoms in sea bass, lowering viral loads in the brain, the primary target tissue. In vitro assays also confirmed that peptide P4, the most promising candidate, partially decreased progeny establishment, likely through immune stimulation, without affecting viral absorption. These findings represent progress toward NNV therapies, highlighting synthetic peptides as promising antivirals, though full protection requires further optimisation.

## Material and Methods

### Bioinformatics Analysis and Structure Modelling

The primary sequence for the capsid protein of the red spotted grouper nervous necrosis virus (RGNNV; acc. number AAZ23225) was obtained from NCBI (https://www.ncbi.nlm.nih.gov/), and secondary and tertiary structures were predicted through the Swiss-Model from Expasy platform (https://swissmodel.expasy.org/). The visualization was made using SwissPdb Viewer (https://spdbv.unil.ch/) (Kaplan and Littlejohn 2001) and Chimera (Pettersen et al. 2004). Structural assessment of the model was made through PDBSum (https://www.ebi.ac.uk/thornton-srv/databases/pdbsum/index.html) (Wiederstein and Sippl 2007; Laskowski et al. 2018). In silico antiviral activity was predicted by iAMPred (http://cabgrid.res.in:8080/amppred/index.html) and AVPred (http://crdd.osdd.net/servers/avppred/) bioinformatics tools (Meher et al. 2017; Thakur et al. 2012).

### Peptide Design and Synthesis 

Peptides, named P1-P7, were synthesized as elsewhere (Guzmán et al. 2007) using solid-phase multiple peptide synthesis with Fmoc amino acids (Iris, Rink resin 0.65 meq/g) and the “tea-bag” method. Cleavage was done with TFA/TIS/H₂O (95:2.5:2.5, Novabiochem), followed by purification via RP-HPLC using a 0–70% acetonitrile gradient (30 min, 1 mL/min). Peptides were lyophilized and their molecular mass confirmed by ESI–MS or MALDI-TOFF (Table 1, Supplementary Data S1). Final products were resuspended in sterile ultrapure water (10 mg/mL) and stored at − 20 °C.

**Table 1:** Peptide sequences designed and synthetized for this study

Peptide	Sequence	Capsid protein position (NH_2_ → COOH)	Location (Domain)	Amino acid conservation (%)	Length (aa)	Molecular weight (Da)
P1	TTKAANPQPRRRANNRRRS	14 — 32	N-arm	84.0	19	2249.52
P2	VSKASTVTGFGR	39 — 50	N-arm	90.0	12	1208.36
P3	PRLGHAARIFQRYA	90 — 103	S-domain	100.0	14	1654.93
P4	PDPTDNDHTFD	129 — 139	S-domain	100.0	11	1272.23
P5	ALQATRGAVVAKWW	140 — 153	S-domain	100.0	14	1555.83
P6	LLWTSSGKEQR	166 — 176	S-domain	90.9	11	1303.47
P7	SVRLSVPSLETPEETTA	204 — 220	Linker region	94.0	17	1815.00

Sequences location according to Chen et al. 2015

### Virus, Cells and Animals

Parental RGNNV (strain It/411/96), SJNNV (strain SJNag97), and reassortant isolates (RGNNV/SJNNV, 367.2.2005; SJNNV/RGNNV, 389/I96) were used. Viruses were propagated in E − 11 cells cultured in L-15 Leibovitz medium (BioWest) supplemented with 5% FBS, 2 mM L-glutamine, and 100 IU/mL penicillin/streptomycin (Thermo Fisher Scientific) at 25 °C. Infected monolayers were incubated at 25 °C until extensive cytopathic effect (CPE) developed. Supernatants were then harvested, centrifuged, and titrated in triplicate using the end-point dilution method, expressed as TCID₅₀/mL (Reed and Müench 1938).

Juvenile sea bass (4.7 ± 0.3 g) were obtained from a hatchery (Cooke España, Cooke Aquaculture Inc.) and maintained in recirculatory aquaculture systems (28% salinity, 25 °C, 12 h light:12 h dark) with appropriate aeration and filtration. Fish were fed daily with a commercial diet (Skretting). All handling complied with EU Directive 2010/63/UE and was approved by the University of Murcia Bioethical Committees (REGA ES300305440012; Permit A13210701).

### In vitro Cytotoxicity and Antiviral Assays

Synthetic peptides cytotoxicity on E − 11 cells was assessed using the MTT (Sigma) test with serial peptide dilutions (1000–4 µg/mL in L − 15 medium; *n* = 3; 24 h at 25 °C) as elsewhere (Cervera et al. 2024c). Absorbance was measured at 570 nm (BMG Labtech); blanks lacked cells. Cell viability was calculated as the OD ratio of treated to untreated cells × 100, normalized at 690 nm.

For in vitro antiviral assays, viral cultures (MOI 0.01) from parental and reassortant strains were incubated with peptides (single or combined) using a non-lethal concentration previously tested (500 μg/mL; León et al. 2020), for 15 min at 25 °C with gentle agitation. Controls were virus samples incubated with PBS (100% viral activity). Treated samples were titrated as previously. After medium removal, cells were exposed to ten-fold viral dilutions in 2% FBS. CPE was monitored daily for 10 days, and viral titres were calculated as TCID₅₀/mL (Reed and Müench 1938) and expressed as viral titre or percent inhibition relative to controls.

Based on in vivo results (see below), the P4 effect on RGNNV replication was further assessed by analysing viral adsorption efficiency and progeny production, as previously (Valero et al. 2021b) with slight modifications. Intracellular and extracellular viral kinetics were examined using semi-confluent E − 11 monolayers in 12-well plates (Sarstedt) treated with P4 (500 µg/mL) for 24 h; untreated cells served as controls. After treatment, supernatants were removed, cells washed thrice, and infected with RGNNV (MOI 0.01) for 45 min. Residual inoculum was collected, and monolayers washed and overlaid with L-15 for incubation at 25 °C with daily CPE monitoring. Supernatants were collected at 8, 12, 24, and 48 h post-infection (hpi); cells were washed, scraped and stored. For extracellular kinetics, supernatants were collected until 168 hpi. All samples were clarified by centrifugation (2000 × g, 10 min). Titrations were performed for: (i) total inoculated virus (TIV), (ii) non-attached virus (NAV), and (iii) viral progeny (VP). Assays were conducted in triplicate.

### RGNNV In vivo Challenge of European Sea Bass

A total of 375 sea bass were randomly assigned to eight experimental groups (in duplicate) and acclimated for 15 days. Seven groups were infected by immersion (10 L; 3.16 × 10^4^ TCID₅₀ RGNNV/mL; 2 h), while a mock-infected control received conditioned medium. Post-infection, fish were immediately intramuscularly injected with either 0.01 M PBS (control/mock) or peptides (P2, P3, P4, P7, P3 + P4, P5 + P6; 1 µg/g). Combinations were selected according to the results obtained in the competitive in vitro assay against RGNNV (see below). Disease signs and mortalities were monitored daily for 23 days post-infection (dpi) using a four-rank scale (Valero et al. 2021a). Fish (*n* = 6/group/time-point) were sampled at 2 and 23 dpi, euthanized with clove oil, bled, and weighed. Brain, NNV target tissue, and head-kidney, major hematopoietic tissue in fish, were dissected, frozen in TRIzol (Life Technologies), and stored at − 80 °C.

### Gene Expression Analysis

For viral load analysis, total RNA was extracted from sea bass brain and head-kidney using TRIzol (Life Technologies) and from E − 11 cells using the GenJET RNA Purification Kit (Thermo Fisher Scientific), following manufacturer protocols. One microgram of RNA was DNAse I-treated (1 U/µL; Thermo Scientific), and cDNA was synthesized using Superscript IV Reverse Transcriptase (Invitrogen). qPCR was performed on a QuantStudio 5 Flex (Applied Biosystems) to quantify *cp*, *rdrp* and *mx* genes using PowerUp SYBR Green reagents (Applied Biosystems). The protocol included a 95 °C hold, then 40 cycles (95 °C; 15 s) and 60 °C for 1 min. Relative expression was calculated using the 2^⁻ΔCt^ method (Livak and Schmittgen 2001), with *ef1a* as the endogenous control. Primers (Supplementary Data S2) were validated by melting curve analysis and negative controls.

### Calculations and Statistics

Formulas used for impact of synthetic peptides on RGNNV replication in vitro assay are the following:

- $$Absorbed\;virus \left(AV\right)=TIV-NAV$$
- $$Adsorption\;efficacy \left(AE\right)=\frac{AV}{TIV}\times 100$$
- $$Production\;rate (PR)=\frac{VP}{AV}$$
- $$Relative\;ratio\;of\;production\;(RRP)=\frac{{PR}_{P4+RGNNV}}{{PR}_{RGNNV}}$$

Fish mortality was analysed using Kaplan–Meier survival curves with log-rank (Mantel–Cox) tests for significance. Relative gene expression was evaluated by one-way ANOVA (*p* ≤ 0.05) followed by Tukey’s test. Viral kinetics were compared between P4-treated and untreated groups using Student’s *t*-test (*p* ≤ 0.05) at specific time points. All analyses were performed with IBM SPSS Statistics 20.

## Results

### Synthetic Peptides Appear to Block the Absorption of Parental and Reassortant NNV Strains In vitro

**Fig. 1:**
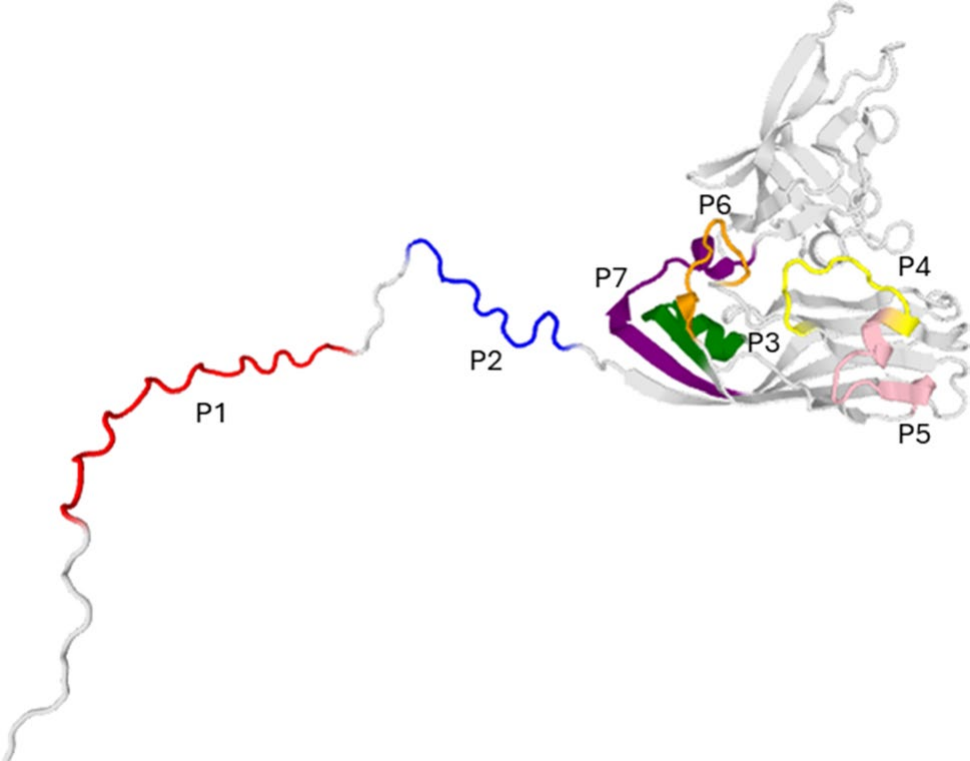
Tridimensional structure of the RGNNV capsid protein under the homotrimeric model highlighting potential binding regions used for the P1 to P7 peptide synthesis. The model was constructed through the SwissModel server and analysed with Chimera as previously described (Cárdenas et al. 2020)

Using the RGNNV capsid protein sequence (AAZ23225), a homology model was generated via SwissModel (Fig. 1). Based on the trimeric capsid model, seven potential contact regions were identified (Table 1, Fig. 1). Peptides corresponding to these regions, P1–P7, were synthesised, ranging from 11 to 19 amino acids and 1208.36 Da (P2) to 2249.52 Da (P1). Results showed that P3, P4 and P5 are in fully conserved regions (100.0% amino acid conservation); while P1, P2, P6 and P7 are highly, but not completely, conserved regions with 84.0%, 90.0%, 90.9% and 94.0% of conserved residues, respectively (Table 1). In silico antiviral activity predictions rated below 0.5 for all peptides (Supplementary Data S3).

**Fig. 2:**
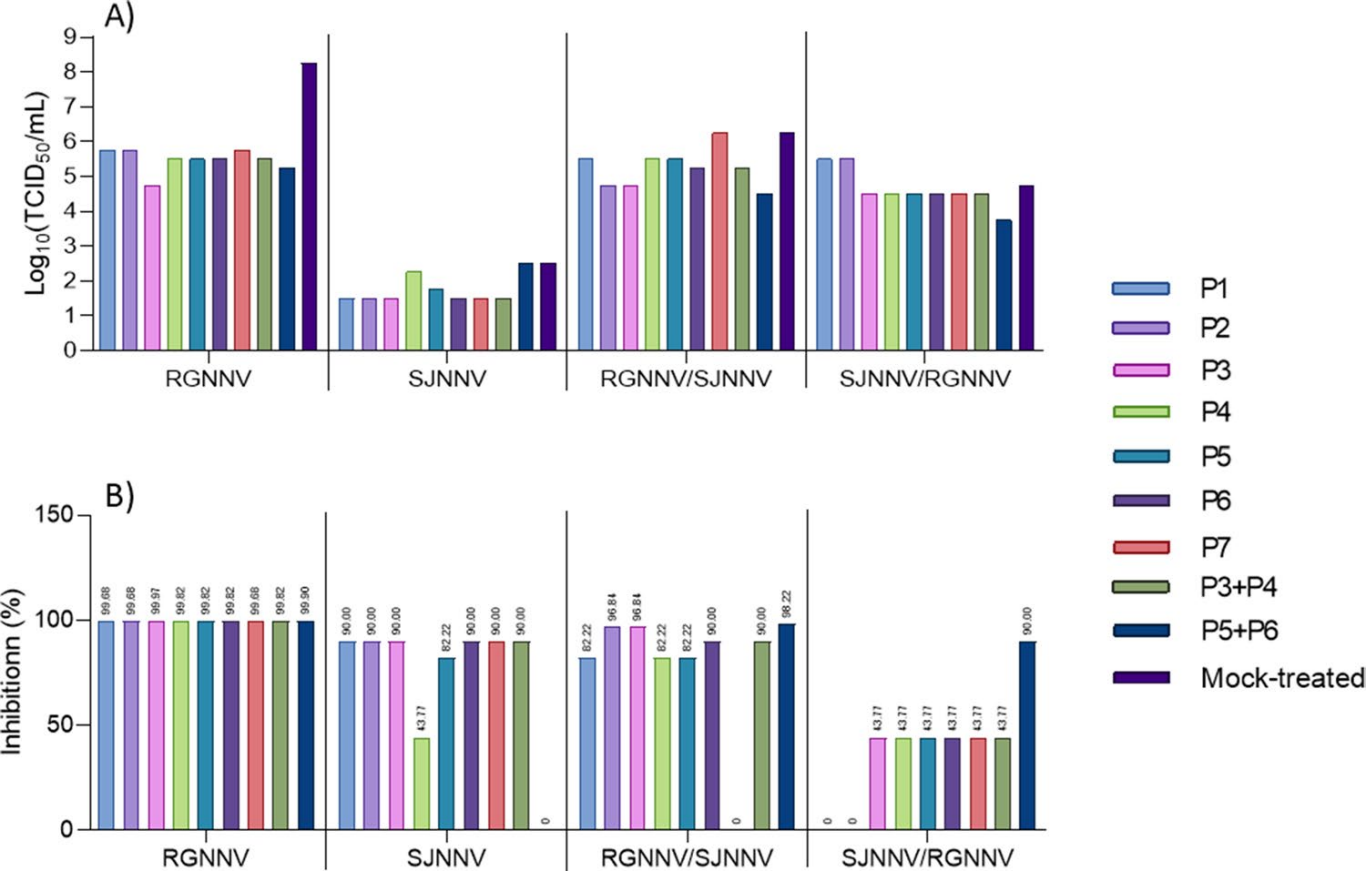
Designed peptides, single or combined, reduce the NNV absorption in vitro. NNV parental or reassortant genotypes were incubated with 500 μg/mL of the synthetic peptides for 15 min. Then, the viruses were titrated in the E − 11 cell line. Control samples consisted on virus incubated with the same amount of phosphate buffer saline. (**A**) Viral titer (TCID_50_/mL) and (**B**) % of virus inhibition with respect to the controls are represented. Data are representative of two independent assays. RGNNV, red-spotted grouper nervous necrosis virus parental strain; SJNNV, striped jack nervous necrosis virus parental strain; RGNNV/SJNNV and SJNNV/RGNNV reassortant strains (RNA1/RNA2 from parental strains)

Antiviral activity of peptides (P1–P7) and combinations (P3 + P4, P5 + P6) was assessed in vitro by their ability to inhibit viral replication when co-applied with the virus (Fig. 2). Peptide safety was confirmed by the absence of cytotoxicity in E − 11 cells (Supplementary Data S4). All peptides and combinations reduced RGNNV titres from ~ 10⁸ to ~ 10^5^–10⁶ TCID₅₀/mL, achieving inhibition of ≥ 99.68% (Fig. 2A, B). Against SJNNV, only P1, P2, P3, P6, P7, and P3 + P4 reached 90% inhibition; others showed lower or no activity (Fig. 2A, B). For reassortant strains, only P2, P3, and P5 + P6 achieved ≥ 90% inhibition (96.84% for P2, P3, and P5 + P6 against RGNNV/SJNNV, and 90% for P5 + P6 against SJNNV/RGNNV) (Fig. 2A, B).

**Fig. 3:**
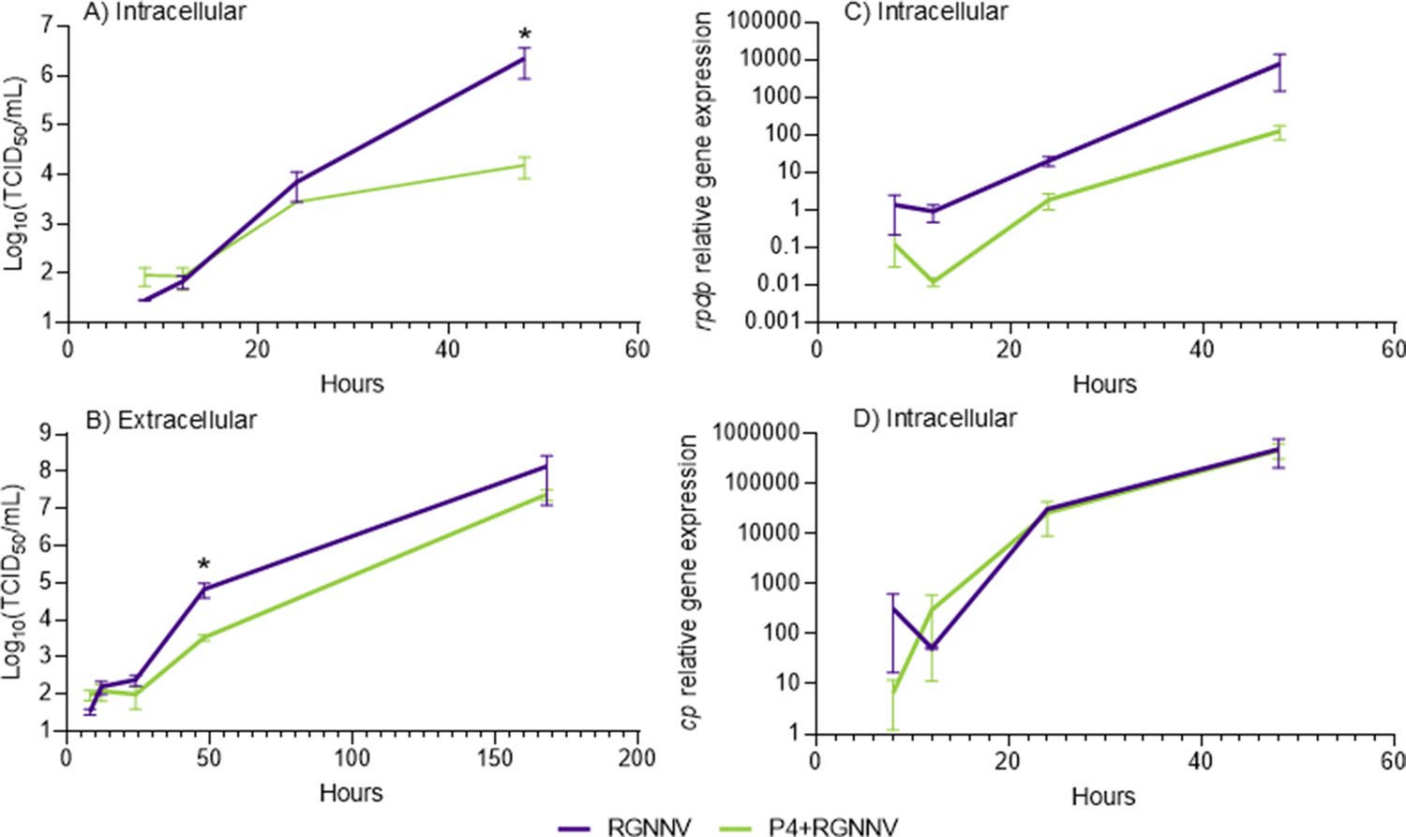
The pre-treatment with P4 synthetic peptide partially disrupts the replication of RGNNV in vitro. (**A**) Intracellular replication kinetics; (**B**) Viral production in cell supernatants; and (**C**, **D**) Genome synthesis in cell lysates by means of transcriptional levels of *rdrp* (C) and *cp* (D) genes. Monolayers of E-11 were incubated with 500 µg/mL of P4 or untreated for 24 h and then infected with red-spotted orange grouper nervous necrosis virus (RGNNV) (*n* = 3). Supernatants and cell lysates were collected at 8, 12, 24, 48 h post-infection (hpi). Additionally, supernatants were sampled at 168 hpi for extracellular replication kinetic analysis. Infectious titers were determined by the end-point titration method (Reed and Müench 1938) and expressed as mean Log_10_ TCID_50_/mL ± standard error of the mean (SEM). Relative expression was presented as the mean ± SEM (*n* = 3). Asterisks indicate significant differences among groups according to the Student-*t* test (*p* < 0.05)

To explore further, we tested peptide P4 based on challenge results (see below). E − 11 cells pre-treated with P4 and then infected showed ~ 99% viral adsorption, similar to untreated cells (9.20 × 10^5^ *vs*. 9.21 × 10^5^ TCID₅₀/mL attached virus; Table 2). However, viral progeny reached 2.46 × 10⁷ TCID₅₀/mL in P4-treated cells *vs* 1.40 × 10⁸ TCID₅₀/mL in untreated cells, with production rates of 26.76 and 151.57, respectively (Table 2). Thus, P4 pre-treatment reduced viral replication, yielding a relative production ratio of 0.18 (Table 2). We measured the production kinetics of infectious particles intra- and extracellularly, along with intracellular viral genome synthesis (Fig. 3). After 48 h, viral replication dropped from 2.40 × 10⁶ to 1.64 × 10^4^ TCID₅₀/mL in untreated and P4-treated cells, respectively (Fig. 3A). Similarly, viral release decreased from 6.77 × 10^4^ to 3.36 × 10^3^ TCID₅₀/mL (Fig. 3B). By day 7, extracellular progeny remained lower with P4, though not significantly (Fig. 3B). Relative *rdrp* gene expression also trended lower with P4, while *cp* gene expression remained similar between groups (Fig. 3C,D).

**Table 2:** Differential replication of RGNNV on mock- or P4-treated E-11 cells

	Viral inoculum (IV)		Attached virus (AV)		AE	Viral progeny (VP)		PR	RRP
	TCID_50_/mL	Log ± SEM	TCID_50_/mL	Log ± SEM		TCID_50_/mL	Log ± SEM		
P4 + RGNNV	9.30 × 10^5^	5.31 ± 0.71	9.21 × 10^5^	5.96 ± 0.005	98.99%	2.46 × 10^7^	7.60 ± 0.87	26.76	0.18
RGNNV	9.30 × 10^5^	5.31 ± 0.71	9.20 × 10^5^	5.96 ± 0.005	99.05%	1.40 × 10^8^	7.35 ± 0.25	151.57	1.00

*AE*, adsorption efficiency; *PR*, production rate; *RRP*, relative ratio of production

### Synthetic Peptides Improve RGNNV-Infected Sea Bass Survival and Reduce Brain Viral Loads without Altering mx Gene Expression in the Head-Kidney

**Fig. 4:**
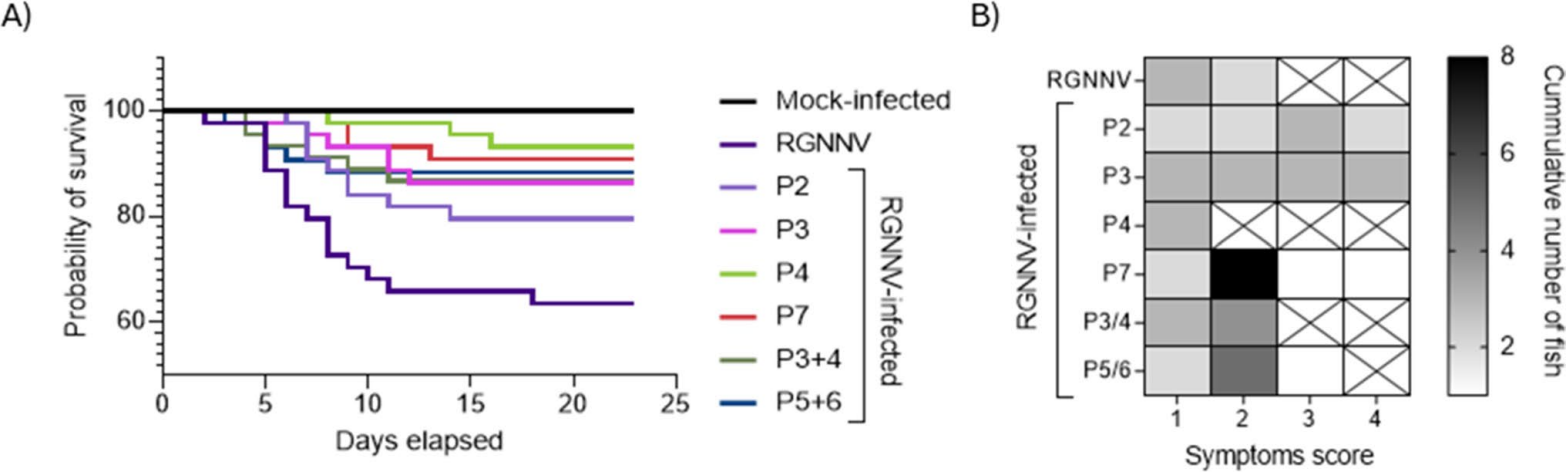
Therapeutic administration of synthetic peptides improves survival and symptomatology in red spotted orange nervous necrosis virus (RGNNV)-infected European sea bass juveniles. Fish were infected by bath with RGNNV (3.16 × 10^4^ TCID₅₀/mL) for 2 h and immediately intramuscularly injected with PBS (RGNNV group) or P2, P3, P4, P7, P3 + P4 or P5 + P6 synthetic peptides (1 μg peptide/g of fish). A mock-infected group was treated twice with only PBS. (**A**) Kaplan–Meier survival curves; (**B**) Heat-map representing the cumulated number of fish showing clinical signs of VER disease attending to their severity: (1) changes of the color of the skin, slower rhythm of swimming and/or slower reaction to external *stimuli* as feeding; (2) alterations in the swimming balance and/or erratic swimming spasms; (3) continuous erratic swimming; and (4) complete incapacity to keep balance, swim and/or move without external *stimuli.* VER, viral encephalopathy and retinopathy

RGNNV-challenged fish showed improved survival and reduced symptoms when treated with synthetic peptides individually or combined after infection (Table 3, Fig. 4A, B). Infected fish had 63.6% survival with modest symptoms before dying (Fig. 4A, B). P4 treatment significantly improved survival (RPS 81.3%) with only minor symptoms (score 1; Table 3, Fig. 4A, B). P7 and P5 + P6 treatments yielded 68.8% RPS but with more severe symptoms, especially with P7 (up to score 4, Table 3, Fig. 4A, B). P3 and P3 + P4 treatments moderately improved survival (RPS 62.5%), though P3 + P4 showed symptoms similar to untreated fish, and P3 alone worsened symptoms (Table 3, Fig. 4A, B). P2 did not significantly improve survival (RPS 43.8%) or reduce symptoms (Table 3, Fig. 4A, B).

**Table 3:** Relative percent survival (RPS) of European sea bass specimens challenged by bath with RGNNV and then injected with the respective peptides. *P* values comparing the respective group with the virus infection according to the Log-Rank test

Group	RPS	Log-Rank test	
P2	43.8	ns	0.0765
P3	62.5	*	*0.0113*
P4	81.3	***	*0.0005*
P7	68.8	**	*0.0016*
P3 + P4	62.5	*	*0.0139*
P5 + P6	68.8	**	*0.0096*

ns, no significant

**Fig. 5:**
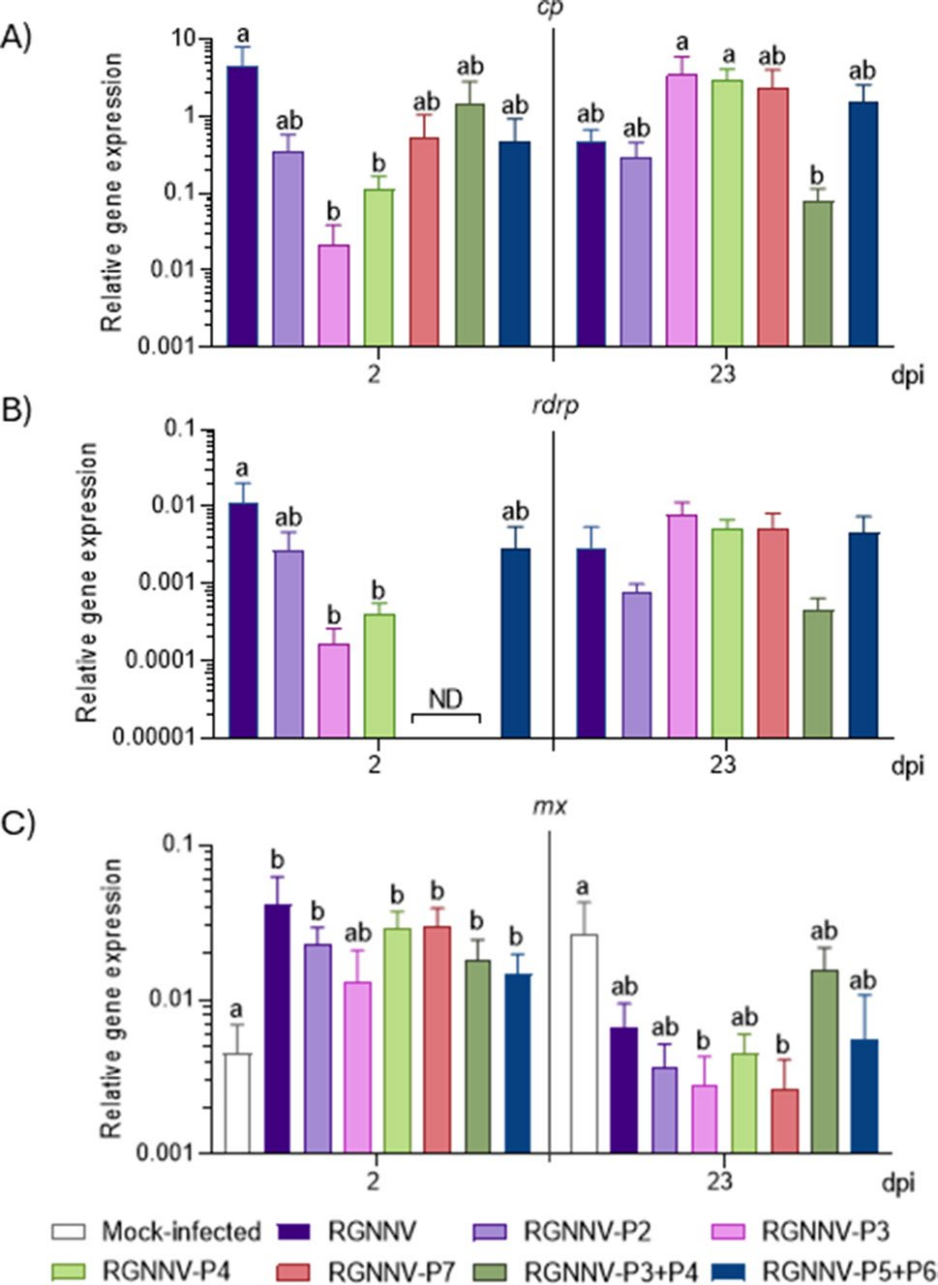
Therapeutic administration of synthetic peptides decreased viral loads in the brain without additional *mx* gene regulation in the sea bass juveniles head-kidney infected with red-spotted grouper nervous necrosis virus (RGNNV). Fish were infected by bath with RGNNV (3.16 × 10^4^ TCID₅₀/mL) for 2 h and immediately intramuscularly injected with PBS (RGNNV group) or P2, P3, P4, P7, P3 + P4 or P5 + P6 synthetic peptides (1 μg peptide/g of fish). A mock-infected group was treated twice with only PBS. (A–C) Transcription levels of RGNNV *cp* (**A**) and *rdrp* (**B**) genes in the brain and (**C**) *mx* gene in the head-kidney. Data represent the relative gene expression mean ± SEM (*n* = 6). Different letters indicate significant differences among groups according to the ANOVA (*p* < 0.05)

Synthetic peptides reduced brain viral load post-infection (Fig. 5A, B). P3 and P4 lowered *cp* and *rdrp* genes expression at 2 dpi, while P7 and P3 + P4 effectively blocked *rdrp* expression (Fig. 5B). Conversely, P2 and P5 + P6 had no transcriptional effect (Fig. 5A, B). Finally, *cp* and *rdrp* genes expression in treated and untreated fish were similar, though the expression of *cp* was notably lower in the P3 + P4 group than with P3 or P4 alone (Fig. 5A). Notably, RGNNV infection, previously treated with peptides or not, significantly triggered the up-regulation of *mx* gene in the sea bass head-kidney after 2 days of infection when compared to the mock-group, except in fish pre-treated with P3 (Fig. 5C). On the contrary, after 23 days of infection, transcription was reduced in all RGNNV-infected groups, reaching significance in those pre-treated with peptides P3 and P7 but not with the RGNNV group (Fig. 5C).

## Discussion

Antiviral peptides, whether naturally derived or synthetically modified, offer a novel, versatile approach in aquaculture by providing targeted antiviral effects, enhancing host immunity, and overcoming the limitations of traditional treatments through customisable, sustainable, and resistance-mitigating strategies (Cárdenas et al. 2020; León et al. 2020). Accordingly, this study designed peptides to disrupt key stages of the RGNNV life cycle. Analysis of the RGNNV capsid protein sequence identified seven potential binding regions capable of interfering with the viral cycle (Chen et al. 2015). If peptides compete with capsid proteins for binding sites—on viral RNA, assembly intermediates, or host factors—they could disrupt host cell attachment or virion assembly (Low et al. 2017). Prediction analyses identified two regions, P1 and P2, within the N-arm (amino acids 1–51); P1 in its disordered portion, while P2 in the ordered segment (Chen et al. 2015). Regions P3 to P6 are located within the S-domain (amino acids 52–213), and P7 in the linker region (Chen et al. 2015). Although the P-domain at the C-terminus governs genotypic differences and virus-host interactions (Chen et al. 2015), no peptides were identified there, likely due to its higher variability in amino acid composition and its unique post-translational modifications (Vimal et al. 2014; Delsert et al. 1997). Specifically, the CP of fish NNV, such as DIEV, appears as a doublet of 43–45 kDa proteins. This size variation is not due to typical proteolytic cleavage or alternative reading frames but is likely the result of non-canonical processing during or shortly after translation (Delsert et al. 1997). Although the exact mechanism and location of this processing are not fully defined, it likely involves the C-terminal region, where the P-domain is located (Chen et al. 2015). This non-standard processing occurs without conserved cleavage sites and may alter the final structure in ways that are not captured by sequence-based prediction tools, limiting the reliability of peptide design for this region. The predicted regions were small, so all were synthesised and purified. In silico antiviral predictions indicated low activity (maximum score of ~ 0.5) for P5 using iAMPred and AVPred, consistent with generally low predicted antiviral AMP activity (Cervera et al. 2024c; León et al. 2020). However, these models, trained solely on amino acid sequences, have recognised limitations for predicting virucidal activity (Charoenkwan et al. 2021).

We tested all peptides and combinations in vitro against various NNV strains using a competition assay in cell lines. Contrary to in silico predictions, all peptides and combinations exhibited over 99% antiviral activity against the RGNNV strain, suggesting interference with viral attachment or entry. Notably, when cells were pre-treated with peptide P4 and infected 24 h later, viral adsorption remained unaffected, but replication and progeny production were limited. The reduction in replication observed 48 h post-infection could reflect host immune responses rather than direct interference, warranting further investigation and aligning with previous in vivo studies. Additionally, our laboratory found that synthetic peptides became undetectable at the administration site after 24 h, yet their immunomodulatory effects persisted (Cervera et al. 2024a). Surprisingly, P1 to P3, P6, P7, and P3 + P4 combination showed 90% antiviral activity against SJNNV, despite a 6–7% capsid sequence divergence between SJNNV and RGNNV (Keawcharoen et al. 2015; Toffolo et al. 2007). The remaining peptides and combinations were less effective, likely because these regions show lower variability than the highly divergent P-domain. Amino acid differences between RGNNV and SJNNV concentrate in the immunogenic P-domain, where critical mutations affect antigenicity and host specificity (Panzarin et al. 2016; Keawcharoen et al. 2015; Souto et al. 2018).

Interestingly, reassortant strains exhibited conflicting tropism patterns. Against RGNNV/SJNNV, which carries SJNNV capsid, P2 and P3 showed over 96.84% antiviral activity, despite lower efficacy against parental SJNNV. Conversely, for SJNNV/RGNNV, bearing RGNNV capsid—the original design target—antiviral effects dropped sharply, often remaining below 43.77%. These results indicate that capsid surface regions and their chemical and electrostatic environments could play critical roles in the viral life cycle of all four genotypes. Notably, structural and chemical differences affecting adsorption can arise even when RNA2 is conserved between parental and reassortant strains. Foundational research on uncoated bi-segmented viruses shows that the RNA1 background influences virion assembly, folding, and stability (Olveira et al. 2009; Eckerle and Ball 2002; Chakravarty et al. 2020), potentially explaining these outcomes. RNA1-encoded polymerase regulates genome replication and capsid assembly and, in reassortants, may alter RNA structures, weaken capsid interactions, and reduce particle stability. In alphanodaviruses, the polymerase also influences capsid formation, membrane dynamics, and subcellular localisation (Bajaj and Banerjee 2016, den Boon et al. 2022, Kopek et al., 2010, Van Wynsberghe and Ahlquist 2009), possibly redirecting assembly to sites like mitochondria and modifying epitope presentation. Understanding these polymerase–capsid interactions is therefore key to assessing reassortant strain stability and antigenicity. Supporting this, studies with neuronal cultures and cell lines from Senegalese sole (*Solea senegalensis*), sea bass, and seabream revealed differing replication kinetics and adsorption rates between RGNNV and its reassortants, despite similar capsids (Souto et al. 2018; Valero et al. 2021b). This suggests RNA1 may influence tropism or receptor engagement by modulating virion structure or entry mechanisms. Additionally, surface charge and hydrophobicity, critical for peptide–virus interactions, depend not only on capsid sequence but also on capsid folding and assembly, both regulated by RNA1(Chen et al. 2015; Olveira et al. 2009). Thus, reassortment may reshape capsid–capsid and capsid–RNA interactions, modifying the electrostatic environment and influencing peptide engagement.

Since co-administered peptides disrupt RGNNV adsorption in vitro, we evaluated their therapeutic potential in vivo by administering them immediately post-infection. Peptides P2, P3, P4, P7, and the combinations P3 + P4 and P5 + P6—selected for their stronger in vitro activity—were tested following a bath challenge in highly susceptible sea bass (LeBreton et al. 1997; Souto et al. 2015). Given RGNNV’s rapid organ colonisation (Valero et al. 2018), treatments were administered 2 h post-infection to ensure viral establishment. Peptides P3 and P4 significantly reduced brain viral loads, as indicated by decreased *cp* and *rpdp* expression at 2 dpi. Peptide P7 and the P3 + P4 combination completely suppressed *rpdp* expression at this time. Interestingly, P4 alone showed more promising results than the P3 + P4 combination, which did not replicate the survival benefit observed with P3 and P4 independently. Peptide-peptide interactions may occur either in vitro and/or in vivo that could interfere with each other’s activity or bioavailability impacting peptide stability, degradation rate and/or structural conformation, ultimately reducing the overall protective effect. This might explain why the P3 + P4 combination was less effective than P4 alone. In parallel, in vitro assays confirmed that P4, the most effective in vivo candidate, substantially reduced intra- and extracellular viral production at 48 h post-infection. This reduction aligned with lower brain viral loads and declining *rpdp* expression, reinforcing the peptides’ antiviral efficacy. These findings suggest that peptides P3–P7 interfere with critical RGNNV life cycle stages, particularly host–virus interactions and adsorption. In fact, in the sea bass head-kidney, only P3 and P7 barely altered the expression pattern of the *mx* gene, the key marker of the type-I interferon pathway, after 23 dpi suggesting that the peptides primarily act as disruptors of host–virus interactions rather than inducing a systemic antiviral state. Likely disrupted processes include genome encapsidation, capsid stability, and integrity at the S–P domain interface (Chen et al. 2015; Štěrbová et al. 2024). Notably, the S-domain, where P4 is located, appears to modulate viral adsorption efficiency, influencing *cp* and *rpdp* transcription and viral entry dynamics. Peptides competing for host binding sites may destabilise capsid–host interactions, impair RNA2 encapsidation, and reduce adsorption efficiency (Chen et al. 2015). Disrupting capsid–capsid interactions has been linked to RNA degradation (Chen et al. 2015), while blocking RNA1 expression may suppress the viral life cycle by limiting capsid protein synthesis or assembly (Low et al. 2017). Despite NNV’s clinical importance, therapeutic studies remain limited and mostly prevention-focused. However, treatments like amantadine improved survival by 44% in RGNNV-infected hybrid grouper (*Epinephelus fuscoguttatus♀* × *E. lanceolatus♂*) (Zhu et al. 2022), while synthetic sea bass peptides Dicentracin and Hepcidin reduced mortality by 30–35%, with Hepcidin also offering strong post-infection anti-inflammatory effects (Cervera et al. 2024b, 2025). Given the short interval between infection and treatment, the tested peptides likely disrupted viral adsorption, apparently without involving a robust antiviral host immune activation, which warrants further investigation.

In conclusion, developing therapies against NNV remains challenging but offers a promising alternative to the limited preventive measures currently available. This study designed peptides targeting the RGNNV life cycle, achieving encouraging in vitro and in vivo results. By competing with capsid protein binding regions, these peptides improved survival and reduced disease symptoms in infected juvenile European sea bass through interference with viral adsorption. They also demonstrated in vitro cross-reactivity against other parental and reassortant genotypes.

## Supplementary Information

Below is the link to the electronic supplementary material.

ESM 1 (DOCX 565 KB)

## Data Availability

No datasets were generated or analysed during the current study.
