# Supplementary material for: Synthetic Peptides Suppress Nervous Necrosis Virus Absorption and Improve Survival Rates in European Sea Bass
Source: Mar Biotechnol (NY). 2025 Aug 23;27(5):128. doi: 10.1007/s10126-025-10507-z (PMC12374861; doi:10.1007/s10126-025-10507-z)
Supplement: Supplementary file 1 — (DOCX 565 KB) [file 10126_2025_10507_MOESM1_ESM.docx]

**Supplementary data S1:** Spectrometry data of the synthesized peptides.

| **RP-HPLC** | **MS** |
| --- | --- |
| **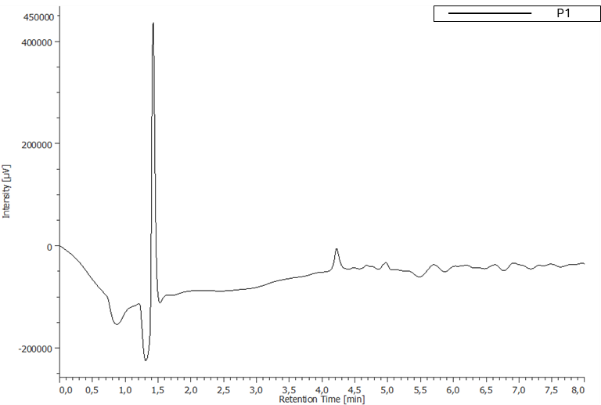** | **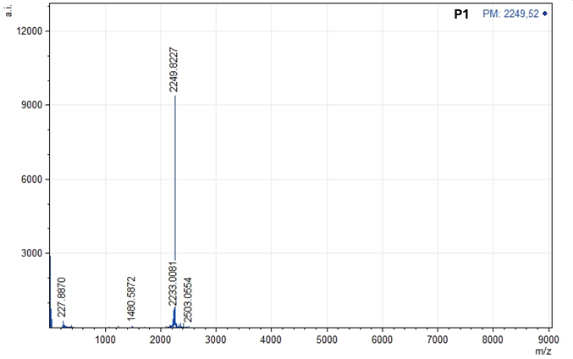** |
| **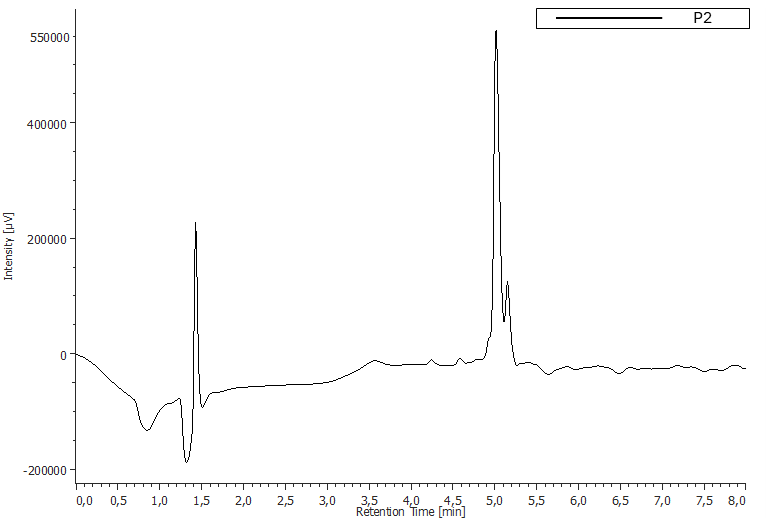** | **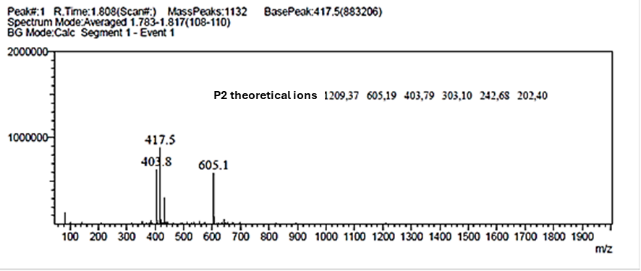** |
| **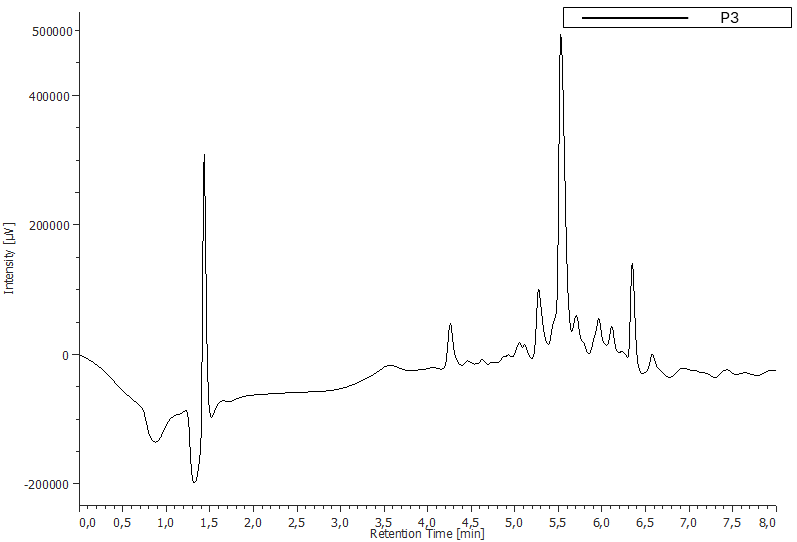** | **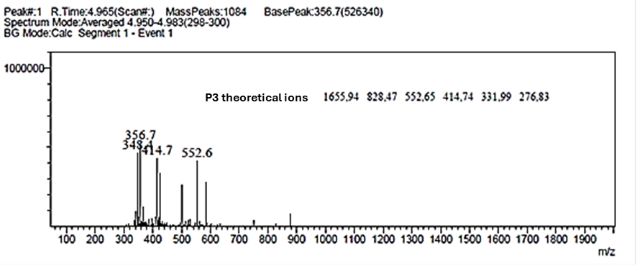** |
| **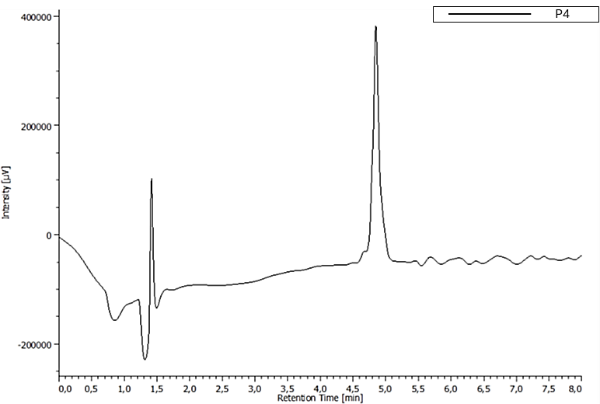** | **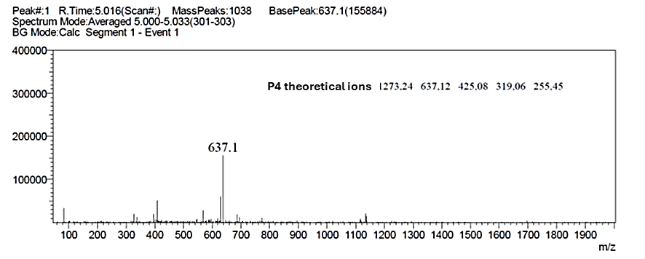** |
| **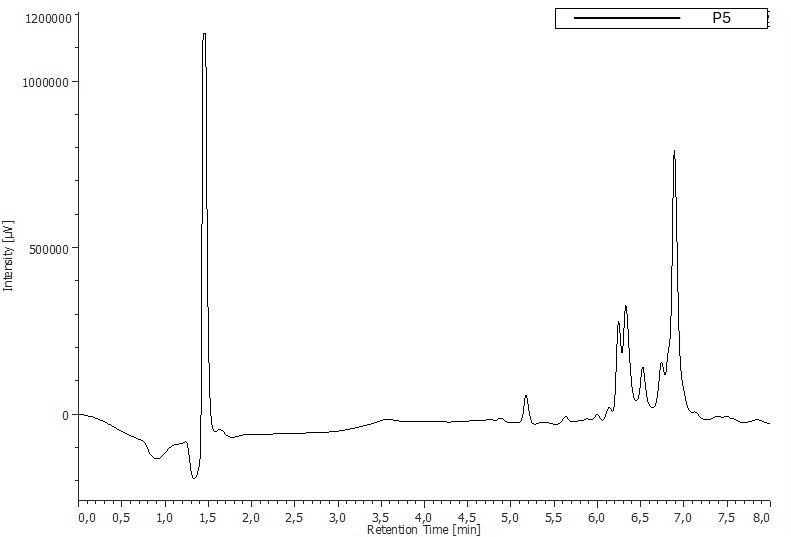** | **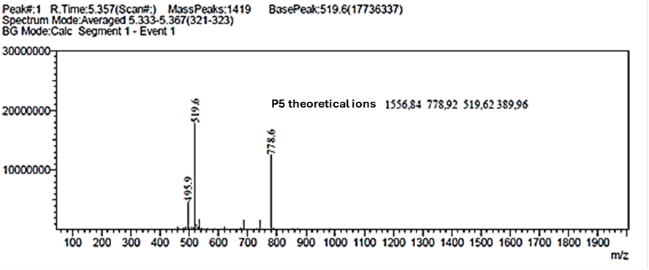** |
| **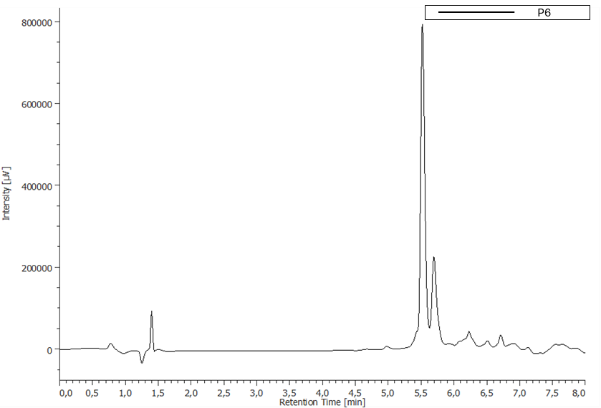** | **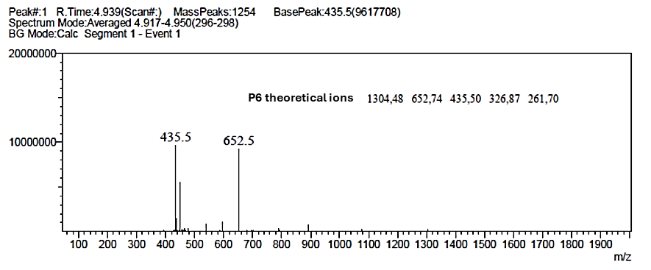** |
| **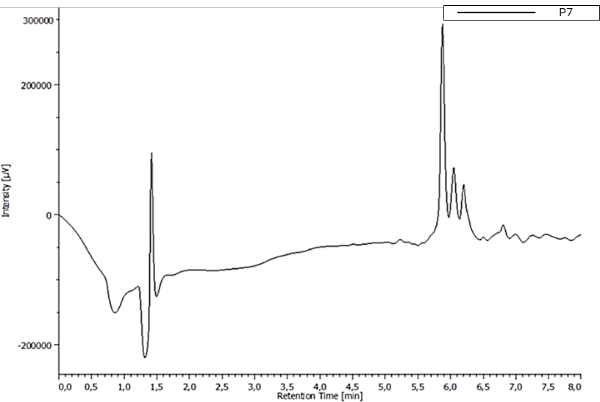** | **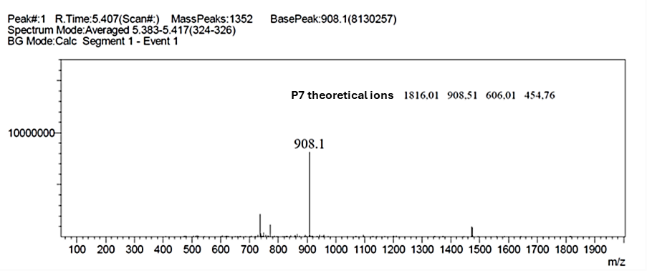** |

**Supplementary data S2:** Primer sequences used in this study.

| **Fish** | **Coding protein** | **Gene** | **Accession number** | **Sequence (5’3’)** | | **Tissue tested** |
| --- | --- | --- | --- | --- | --- | --- |
| European sea bass | Elongation factor 1 alpha | *ef1a* | FM019753 | F | CGTTGGCTTCAACATCAAGA | Brain |
|  |  |  |  | R | GAAGTTGTCTGCTCCCTTGG | Head-kidney |
|  | Interferon-induced GTP-binding protein Mx | *mx* | AM228977 HQ237501 AY424961 | F | GTATGAGGAGAAGGTGCGTCC | Head-kidney |
|  |  |  |  | R | CTCTTCCCCGAGCTTTGGTC |  |
| NNV | Capsid protein | *cp* | D38636 | F | CAACTGACAACGATCACACCTTC | Brain |
|  |  |  |  | R | CAATCGAACACTCCAGCGACA |  |
|  | RNA-dependent RNA polymerase | *rdrp* | AF319555 | F | AAATTGCACACCACCTGTGA | Brain |
|  |  |  |  | R | ACCCAGAATGGAATGTCAGC |  |

**Supplementary data S3:** Predicted *in silico* activity of the studied synthetic peptides against viruses using iAMPred and AVPred tools.

|  | **iAMPred** | **AVPred** |
| --- | --- | --- |
| P1 | 0.087 | 0.395 |
| P2 | 0.033 | 0.295 |
| P3 | 0.291 | 0.417 |
| P4 | 0.355 | 0.421 |
| P5 | 0.521 | 0.497 |
| P6 | 0.132 | 0.479 |
| P7 | 0.018 | 0.355 |

**Supplementary data S4:** Cell viability (%) of E-11 cells after incubation with the synthetic peptides for 24 h. Data represent the mean ± SEM (n=3).

|  | Viability (%) | | | | | | | | | | | | | |  |
| --- | --- | --- | --- | --- | --- | --- | --- | --- | --- | --- | --- | --- | --- | --- | --- |
| Peptide concentration | P1 | | P2 | | P3 | | P4 | | P5 | | P6 | | P7 | | |
| (mg/mL) | Mean | SEM | Mean | SEM | Mean | SEM | Mean | SEM | Mean | SEM | Mean | SEM | Mean | SEM | |
| 1.00000 | 91.5 | 7.562 | 117.2 | 7.055 | 101.7 | 10.708 | 106.4 | 10.560 | 115.8 | 3.014 | 128.1 | 3.252 | 131.6 | 25.349 | |
| 0.33333 | 109.3 | 5.832 | 126.9 | 21.294 | 90.9 | 8.160 | 100.5 | 11.514 | 104.3 | 13.040 | 146.5 | 7.334 | 119.8 | 18.452 | |
| 0.11111 | 96.6 | 12.773 | 106.2 | 6.937 | 89.8 | 1.086 | 110.6 | 19.110 | 122.7 | 14.467 | 152.9 | 7.234 | 116.6 | 17.896 | |
| 0.03704 | 100.1 | 8.100 | 111.5 | 10.806 | 96.3 | 7.613 | 92.4 | 3.828 | 116.4 | 12.380 | 138.9 | 6.370 | 138.9 | 4.155 | |
| 0.01234 | 129.9 | 21.740 | 121.8 | 14.269 | 93.2 | 9.611 | 102.6 | 6.213 | 116.7 | 8.056 | 139.2 | 1.966 | 125.7 | 17.444 | |
| 0.00411 | 123.5 | 10.658 | 103.1 | 4.237 | 89.4 | 5.779 | 87.4 | 3.676 | 126.6 | 16.081 | 157.5 | 8.132 | 149.6 | 14.646 | |
| 0 - Control | 100.0 | 4.982 | 100.0 | 4.982 | 100.0 | 4.982 | 100.0 | 4.982 | 100.0 | 4.982 | 100.0 | 4.982 | 100.0 | 4.982 | |
